# Genome-Wide Analysis of the Synonymous Codon Usage Patterns in *Riemerella anatipestifer*

**DOI:** 10.3390/ijms17081304

**Published:** 2016-08-10

**Authors:** Jibin Liu, Dekang Zhu, Guangpeng Ma, Mafeng Liu, Mingshu Wang, Renyong Jia, Shun Chen, Kunfeng Sun, Qiao Yang, Ying Wu, Xiaoyue Chen, Anchun Cheng

**Affiliations:** 1 Institute of Preventive Veterinary Medicine, Sichuan Agricultural University, Wenjiang, Chengdu 611130, China; eaeas12@163.com (J.L.); liumafengra@163.com (M.L.); cqrc_jry@163.com (R.J.); sophia_cs@163.com (S.C.); 18981613263@163.com (K.S.); yangqiao721521@sina.com (Q.Y.); yingzi_no1@126.com (Y.W.); 2 Key Laboratory of Animal Disease and Human Health of Sichuan Province, Sichuan Agricultural University, Wenjiang, Chengdu 611130, China; zdk24@sicau.edu.cn (D.Z.); chenxy_24@sina.cn (X.C.); 3 China Rural Technology Development Center, Beijing 100045, China; maguangpeng1977@163.com

**Keywords:** *Riemerella anatipestifer*, codon usage bias, natural selection, highly expressed gene

## Abstract

*Riemerella anatipestifer* (RA) belongs to the *Flavobacteriaceae* family and can cause a septicemia disease in poultry. The synonymous codon usage patterns of bacteria reflect a series of evolutionary changes that enable bacteria to improve tolerance of the various environments. We detailed the codon usage patterns of RA isolates from the available 12 sequenced genomes by multiple codon and statistical analysis. Nucleotide compositions and relative synonymous codon usage (RSCU) analysis revealed that A or U ending codons are predominant in RA. Neutrality analysis found no significant correlation between GC_12_ and GC_3_ (*p* > 0.05). Correspondence analysis and ENc-plot results showed that natural selection dominated over mutation in the codon usage bias. The tree of cluster analysis based on RSCU was concordant with dendrogram based on genomic BLAST by neighbor-joining method. By comparative analysis, about 50 highly expressed genes that were orthologs across all 12 strains were found in the top 5% of high CAI value. Based on these CAI values, we infer that RA contains a number of predicted highly expressed coding sequences, involved in transcriptional regulation and metabolism, reflecting their requirement for dealing with diverse environmental conditions. These results provide some useful information on the mechanisms that contribute to codon usage bias and evolution of RA.

## 1. Introduction

*Riemerella anatipestifer* (RA), belonging to the *Flavobacteriaceae* family, is a non-spore-forming, rod-shaped, and atrichous Gram-negative bacterium [1]. It can cause a contagious disease in domestic ducks, geese, turkeys, and various other wild birds. To date, more than 21 serovars have been identified [2]. In addition, no cross-protection has been observed with inactivated bacterins made from different serotypes of RA [3]. Thus, RA can easily cause large economic losses in the duck industry over the world.

Codon usage bias (CUB) of genes generally exists in prokaryotes and eukaryotes. The genetic code in organisms is not strictly one-for-one code. Most amino acids, except Trp (UGG) and Met (ATG) can allow more than one codon (called synonymous codon). Synonymous codons usually differ by one base in the third codon position (or for some amino acids, in the second position) [4,5]. Among prokaryotes, it is well known that CUB is mainly influenced by mutational bias and natural selection [6,7]. Mutational bias can drive the change in the G + C content of the whole genome. Examples of mutational bias affecting codon usage can be illustrated in many prokaryotes with extremely AT or GC-rich genome [8]. Moreover, CUB may be associated with some other factors, including gene expression level [9,10], gene length [11], amino acid conservation, protein structure [12], gene function [13], and isoaccepting tRNA [14]. There are some variations in codon usage among the genomes of bacteria, which suggests that these genomes bear different pressure in evolution process. CUB analysis has important significance in many aspects. It was proved useful in studying molecular genetic engineering for codon optimization and heterologous protein expression in some species [15]. CUB analysis at genomic scale can also reveal the genetic information about the molecular evolution of individual genes and help to understand evolution of living organisms [16]. Furthermore, CUB can enrich our understanding about the relationship between pathogens and their hosts by analyzing their codon usage patterns [17].

At present, CUB in RA has not been investigated in any detail, and is not clear which factors shape the codon usage pattern. In this study, we analyzed the genome-wide codon usage patterns of 12 RA species. Our results show that natural selection is the main driving factor for codon usage patterns of RA. Additionally, the evolutionary relationship of the species shown in our study is different from that of the traditional classification.

## 2. Results

### 2.1. The Codon Usage Pattern between Riemerella anatipestifer (RA)

**Figure 1:**
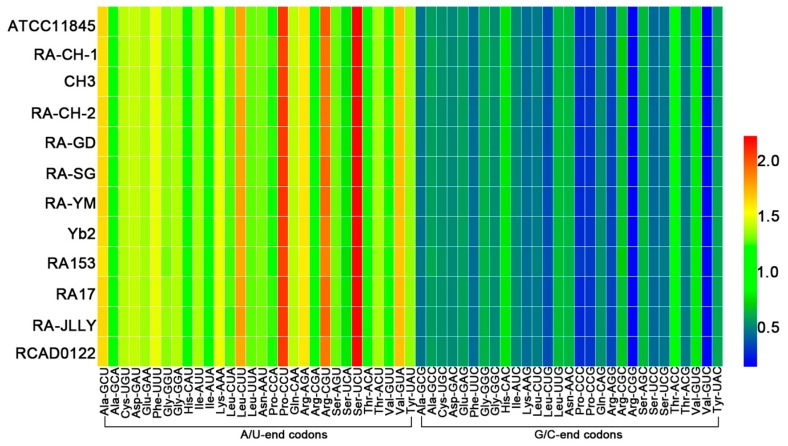
Comparison of RSCU between 12 different species of RA. The heat-map was drawn by HemI using hierarchical clustering method. The higher RSCU value, suggesting more frequent codon usage, was represented with darker shades of red. In *Riemerella anatipestifer* (RA) genomes, codons ending in A or U have higher RSCU value than codons ending in G or C.

To identify and understand codon usage patterns of RA, the values of relative synonymous codon usage (RSCU) were computed for every codon in each genome. A codon with an RSCU value of more than 1.0 has a positive codon usage bias, while a value of less than 1.0 has a negative codon usage bias. When the RSCU value is equal to 1.0, it means that this codon is chosen equally and randomly [18]. The results showed a general bias toward codons having nucleotides A or U in the third position while U was more frequently detected (Figure 1 and Table 1). There were 30 codons having the high RSCU values (RSCU > 1, Table 1; in bold), and optimal codons (shown in *, Table 1) identified by χ-squared test which were similarly biased. Among the RA strains, it can be clearly observed that the frequencies of UCU (Ser) and CCU (Pro) are considerably high.

### 2.2. The Codon Usage Bias of RA not Affected by Mutation Bias

The preference of A or U in the third position of the codon in RA observed in the RSCU comparative analysis could be due to the overall GC bias within the genome. Differences in GC content were the greatest at the third codon position followed by the first and second positions [19]. The GC3s values of RA strains varied from 27.07% to 26.50% with a mean of 26.6% and standard deviation (SD) of 0.23. The effective number of codons (ENc) has been widely used to measure the codon bias level of individual genes. Among the 12 isolates, the values of ENc were higher than 40 (Table 2) ranging from 45.04 and 45.47. With the mean value of 45.20 and S.D. of 0.16 (*p* > 0.05), this indicates that CUB has no bias in RA genomes.

**Figure 2:**
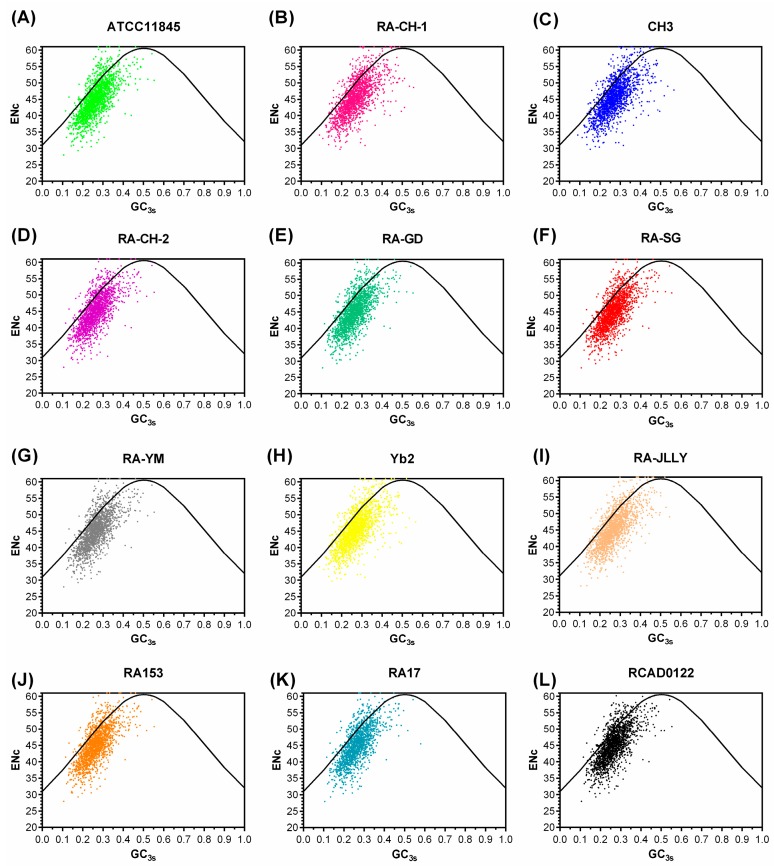
The ENc vs. GC3s plots of RA genomes. (**A**) ATTCC11845; (**B**) RA-CH-1; (**C**) CH3; (**D**) RA-CH-2; (**E**) RA-GD; (**F**) RA-SG; (**G**) RA-YM; (**H**) Yb2; (**I**) RA-JLLY; (**J**) RA153; (**K**) RA17; and (**L**) RCAD0122. The standard curve represents the expected ENc to GC3s. Most RA genes are far away from the standard curve, showing that their codon usage pattern might be affected by other factors besides nucleotide composition. Some genes with the ENc score of 61 display no bias and use all the 61 sense codons.

Plotting ENc versus GC3s is an effective strategy to investigate patterns of synonymous codon usage [20]. The distribution plot of ENc and GC3s values for these genes have been presented in Figure 2. The solid line represents the curve if codon usage is only determined by GC3s. The actual ENc values for some genes lay near to the solid line on the left region of this distribution, and a majority of the points with low ENc values lay below the expected curve. This implies that not only mutation but also other factors are likely to be involved in determining the selective constraints on codon bias in RA genomes.

### 2.3. Correspondence Analysis (COA)

**Figure 3:**
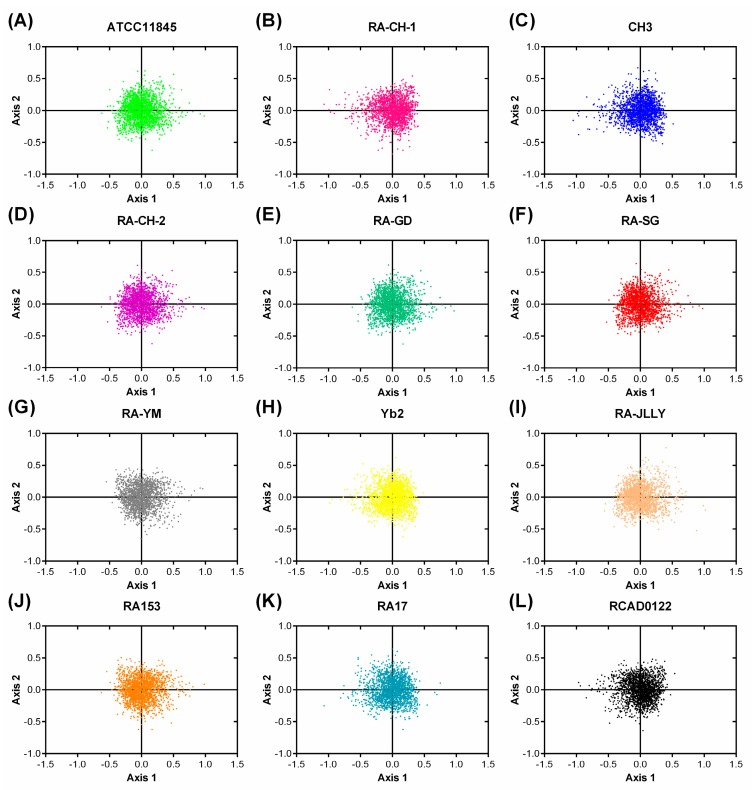
The correspondence analysis (COA) of the genes in RA genomes. (**A**) ATTCC11845; (**B**) RA-CH-1; (**C**) CH3; (**D**) RA-CH-2; (**E**) RA-GD; (**F**) RA-SG; (**G**) RA-YM; (**H**) Yb2; (**I**) RA-JLLY; (**J**) RA153; (**K**) RA17; and (**L**) RCAD0122. Each point represents a gene corresponding to the coordinates of the first and second axes of variation generated from the correspondence analysis.

To investigate the synonymous codon usage variation among RA strains, COA was performed on the variation of RSCU value for this study. The coordinate of each coding sequence (CDS) on the two principal axes (Axes 1 and 2) is shown in Figure 3. The relative inertia explained by the first axis in RA contributes approximately 10% of the total variation. It must be remembered that although the first principal axis explains a substantial amount of variation of codon usage among the genes in RA, its value is not remarkably high for relative inertia explained by the first axis in other organisms studied earlier [11,21,22]. The low value might be due to the AT-rich genomic composition of this genome. As it can be seen, these strains of RA isolated from different places, even the same serotype, have the same trend in codon usage variation. The previous studies have shown that the codon usage variation among the genes in the extremely AT or GC rich organisms is only shaped by compositional bias, The third codon position in the preferred codons should also have the base composition of A or T [23,24]. The mutation bias toward a high G + C content seems to have resulted in a preponderance of GC-rich optimal codons [25]. As shown in Table 1, the third positions of optimal codons in RA were preferred in A or T, which suggests that the strongest influence on the choice of codon usage might not be mutation bias, but translation optimality in RA.

### 2.4. Natural Selection Influences the Codon Bias of RA 

**Figure 4:**
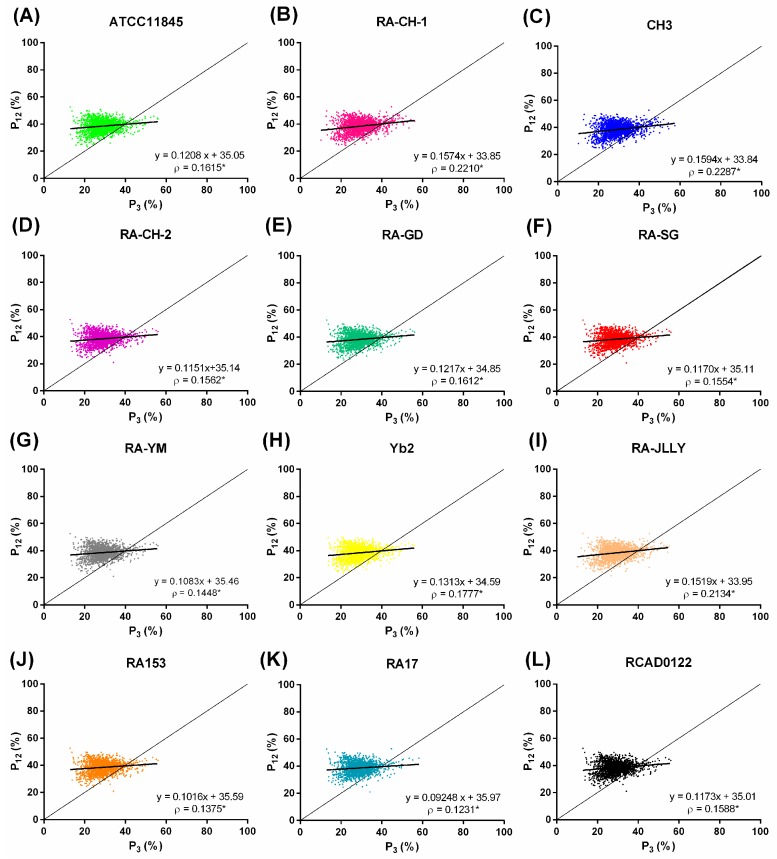
Neutrality plots of RA genomes. (**A**) ATTCC11845; (**B**) RA-CH-1; (**C**) CH3; (**D**) RA-CH-2; (**E**) RA-GD; (**F**) RA-SG; (**G**) RA-YM; (**H**) Yb2; (**I**) RA-JLLY; (**J**) RA153; (**K**) RA17; and (**L**) RCAD0122. Individual genes are plotted based on the mean GC content in the first and second codon position (P_12_) versus the GC content of the third codon position (P_3_). Regression lines and Spearman’s rank correlation coefficients (ρ) are shown, with the asterisk (*) denoting *p*-values < 0.01.

The GC content is calculated according to the first, second, and third codon positions (P_1_, P_2_ and P_3_ respectively). P_12_ is the average of P_1_ and P_2_, it is used for analysis of neutrality plot (P_12_ against P_3_). The neutrality plot is drawn to characterize the correlation among the three codon positions, and then used to estimate the extent of directional mutation pressure against selection on CUB. In the neutrality plot, each point represents one gene (Figure 4). If a gene is under neutral selection pressure, a point should be located on diagonal line with a significant correlation between its P_12_ and P_3_. If a gene is close to *X*-axis, below the diagonal line, meaning the gene is under mutational pressure. Thus, the slope less than 1 should indicate a whole genome trend of non-neutral mutational pressure [26,27]. In this study, all RA species had relative neutralities ranging from 9% to 15% (Figure 4). It means CUB was affected a little by neutral evolution since natural selection was more than 85%. The points in all RA species were located above the diagonal distribution and the regression curve (bold line) with a slope less than 1, indicated the whole genomes in RA species trend of non-neutral mutational pressure. The subsequent correlation analysis revealed little positive correlation between P_12_ and P_3_. These results showed that natural selective pressure dominated over mutation shaping the composition of coding sequences.

### 2.5. Cluster Analysis

**Figure 5:**
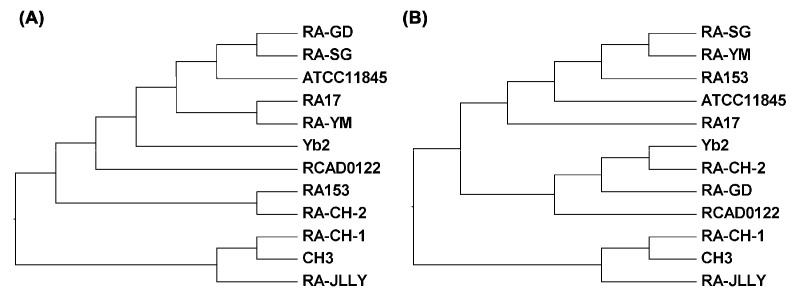
Comparison of phylogenetic tree with RSCU based clustering of RA strains. (**A**) The phylogenetic tree derived for the RA genome using genomic BLAST by neighbor-joining method; (**B**) Cluster analysis of the 12 species in RA based on RSCU value. The observed distances range from 1 to 25, the ratio of the rescaled distances within the dendrogram is the same as the ratio of the original Squared Euclidean distances.

To gain more insight into evolution of the RA, the RSCU values between 12 species were used in hierarchical clustering (Figure 5B). Cluster analysis for RA family yielded five major clusters, similar to dendrogram based on genomic BLAST by neighbor-joining method (Figure 5A). Cluster I is composed of RA-CH-2, RA-GD, Yb2 and RCAD0122, meanwhile RA-CH-2 and Yb2 stay the closest and are isolated almost from the root. Cluster II contains ATCC11845, RA153, RA-SG and RA-YM. RA-SG and RA-YM appear closely related to RA153, but are on different branches compared to ATCC11845. RA17 has a close relationship with cluster II divided into cluster III. RA-CH-1 and CH3, belonging to the serotype 1, are clustered in cluster IV. The highly biased RA-JLLY is clustered alone as a minor cluster of cluster V and close to the branch of RA-CH-1 and CH3.

### 2.6. Understanding Pathway Level Functions in RA through CUB

**Figure 6:**
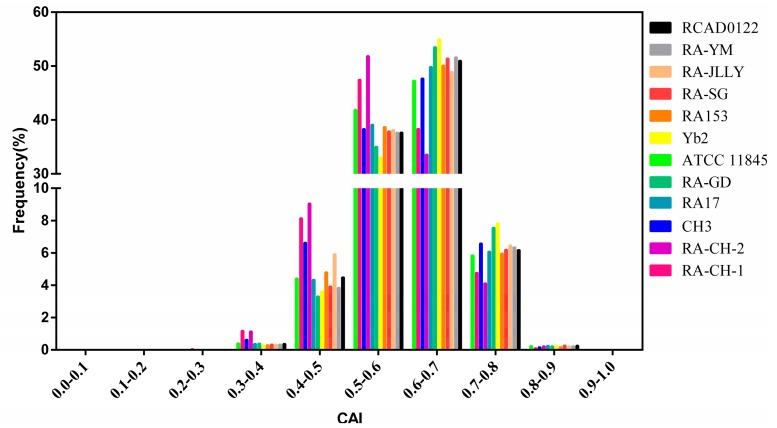
Frequency distribution of codon adaptation index (CAI) values for coding sequence (CDS) in the genomes of RA strains.

The codon adaptation index (CAI) for a gene is a measurement of its optimal codons usage, which is the codon commonly used by highly expressed proteins in a given genome [28]. CAI values of all CDS in RA genomes were calculated using the ribosomal protein codon usages as a reference set. As shown in Figure 6, the CAI values of all RA genes were distributed over a very wide range from 0.3 to 0.8 (the mean value of 0.6), but most of the genes had CAI values between 0.5 and 0.7. Only about 6% of the CAI values were greater than or equal to 0.7. No obvious correlation was observed between CAI values and the corresponding gene lengths (*p* > 0.05). This implies that codon bias is not the primary mechanism determining the translational efficiency of long genes in RA. Within each RA strain, the top 5% of genes with the highest CAI value were predicted to be highly expressed genes. This is corresponded to CAI cut-off of 0.701 in ATCC11845, 0.698 in RA-CH-1, 0.708 in CH3, 0.691 in RA-CH-2, 0.709 in RA-GD, 0.706 in RA-SG, 0.706 in RA-YM, 0.715 in Yb2, 0.708 in RA-JLLY, 0.703 in RA153, 0.700 in RA17, and 0.706 in RCAD0122 (included about 100 genes for each RA strains), respectively.

To further analyze the highly expressed genes estimated by functional analysis, we used blastKOALA based on KEGG annotations [29]. As the limitation of gene annotation and functional studies, about forty-five orthologous high-level expression gene pairs from the all RA genomes were annotated as hypothetical proteins. In this way, their CAI values could rightfully indicate the gene expression level. These hypothetical proteins with the predicted high expression may become attractive candidates for experimental characterization, thus we assumed that they should have important functions in those organisms. Functional analysis showed that only half of genes in all 12 genomes were classified. The high-level expression genes were involved in genetic information processing, carbohydrate metabolism, energy metabolism, metabolism of cofactors and vitamins, nucleotide metabolism and cellular processes (Table 3). The high-level expression genes involved in genetic information processing were the largest functional group. An investigation of the functional categories to which the CAI reference genes (top 1% of genes) belong has revealed that RA contains a significant fraction of ribosomal proteins (large subunit ribosomal in 62.5% and small ribosomal subunits in 37.5%). This is in agreement with the ribosomal criterion defined by Carbone [30], which states that ribosomal proteins have significantly higher CAI value than other protein encoding genes in translationally biased organisms. The *rplL* encoding ribosomal protein L9 with the highest CAI value (0.834) was one of the most abundant proteins under the rapid growth conditions in RA while codon selection was expected to be effective. The second most high-level expression genes was for various enzymes including carbohydrate metabolism, metabolism of cofactors and vitamins, energy metabolism and nucleotide metabolism. As we know, *acnA*, *mdh*, *sucC* and *sucD* gene encoding aconitate hydratase, malate dehydrogenase and succinyl-CoA synthetase are participant in tricarboxylic acid cycle (TCA) pathway. Several genes encoding cytochrome, transferases, and ATP synthase were also found in the 12 RA strains. Enolase is involved in secondary metabolism. Apart from ribosomal proteins and enzymes, three genes encoding elongation factor Tu, G, Ts and two chaperone encoding GroEL and DnaK were observed as the high-level expression genes in RA genomes. In addition, the outer membrane protein was also found high in expression. This analysis has offered the prospective method to further carry out the characterization on those genes.

## 3. Discussion

To confirm the observed dominance of mutational bias, the RSCU patterns are conducted in these strains. As a general rule, AT-rich genome of bacteria can result in the dominance of the A/U-ended codons. RA has extremely AT-rich genome, which is the main reason why there are 31 optimized codons ending with A/U among 32 optimized codons. This predominance of A and T at the synonymous sites is better displayed in Table 2, which reveals that amino acid usage is strongly associated with AT content in AT-rich genome [31]. In bacteria with extreme genomic GC compositions, synonymous codon usage could be dominated by strong compositional bias [32].What is more, the mutation is universally biased towards AT in bacteria [33,34]. Therefore, it likely can be concluded that the main force driving codon usage in RA is the strong compositional bias towards A and T. It is reasonable that compositional bias may be a potential bias in the evolution of the codons in RA.

The codon usage bias was conserved in RA strains. The RSCUs of each codon were very similar in 12 RA strains. Meanwhile, the distributions of the plot of Axes 1 and 2 in each CDS were almost in the same region. The plot of Axes 1 and 2 of each open reading frame (ORF) shows that there is a quite small amount of the codon usage variation in RA strains. In addition, the COA also has highly negative correlation with the GC3s value, which suggests codon usage variation is directly related to mutational bias. The ENc values of RA genome are all more than 45, which demonstrates that codon usage bias is low in RA strains. The ENc-plot suggests that not only mutational pressure but also other factors affect the codon bias among the genes. This conclusion is also supported by the highly significant correlation analysis. Comparisons of 12 RA species show a significant positive correlation between ENc and GC3s (*p* < 0.01). Moreover, it is obviously that the codon usage bias has no significant difference by comparing the ENc-plot of 12 RA strains. In summary, the data presented herein reveals that the differences of codon usage are small among different RA strains.

Most CAI values of RA genes are near to 0.6 that is lower than other bacteria, such as *E. coli*, *Nocardia farcinica*, and *Streptomyces coelicolor* [35,36,37]. The results provide evidence why RA strains need rich nutrition to grow but still slow and consequently have low environment adaptability. By correlation analysis between average RSCU values of RA ORFs and high/low ENc value groups, there are high correlations between RA ORFs and high/low ENc value groups. The codon usage patterns have no obvious difference between high and low ENc value genes. Hence, gene expression levels only have a weak influence on codon usage bias in RA.

Finally, the CAI values were set as the expression level indicator of genes in RA. The notion of gene expression by CAI values was proposed for a long time ago, however, in recent years, the methods have been widely used to qualitatively assess high-level expression genes in prokaryote and eukaryote [38,39,40,41]. Fast development of the whole-genome analysis technologies, especially whole genome sequencing as well as proteomics has made it possible to compare computational data of codon usage and expression ability with experimental evidence. In our research, the highly expressed genes can be considered as the strength of relative codon bias, most of the highly expressed genes are identified by ribosomal proteins genes. Moreover, the genes encoding elongation factor, chaperone proteins, enzymes of essential carbon metabolism pathways of TCA cycle, genes of ATP synthesis, nucleotide biosynthesis, outer membrane protein, transport and binding protein are identified as highly expressed genes in our approach. The study also proves our prediction, based on their codon usage, that some of hypothetical proteins would be highly expressed. Further research of hypothetical proteins by integrated computational and experimental data will enhance our knowledge of the metabolism in RA.

## 4. Materials and Methods 

### 4.1. Sequences Data

**Figure 7:**
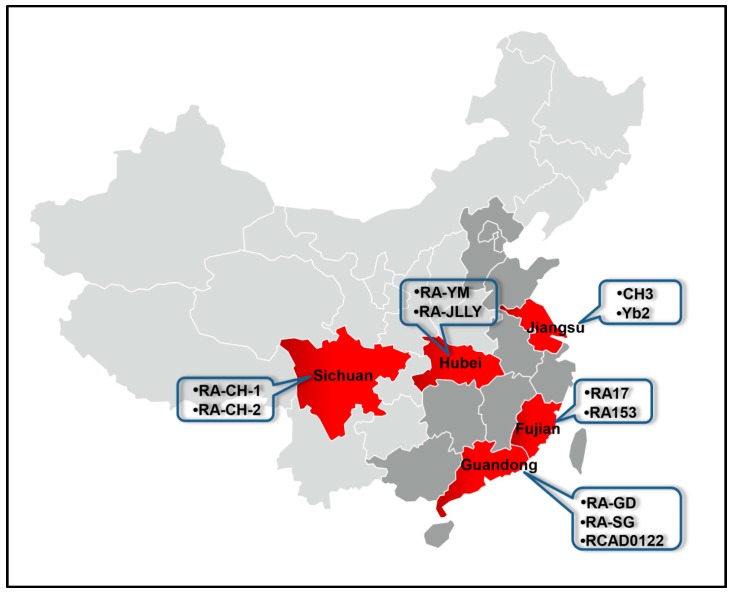
Geographical location of the RA analyzed in this study. The main distribution of duck industries in China are shown in dark gray. The provinces (regions) of the RA strains in this study are indicated in red.

A total of 12 RA genomes were used in this study. The coding sequences (CDS) datasets from the whole genome sequences were obtained from National Center of Biotechnology Information (NCBI). To minimize sampling bias in codon usage calculations only CDS of at least 100 codons in length with correct initiation were used in further analysis. Detailed information about these strains is listed in Table 4, and the distribution of these strains except ATCC11845 in the different provinces of China is shown in Figure 7.

### 4.2. Measurement Indices of Codon Usage Bias

In order to normalize codon usage within datasets of different amino acid compositions, relative synonymous codon usage (RSCU) values were calculated by dividing the observed codon usage by the expected ones under the condition that all codons for the same amino acid are used equally. The RSCU was used to compute relative codon frequency. The codon adaptation index (CAI) has been proved to be the best gene expression value index and was extensively used as a measure of gene expression level. The CAI was generally calculated using the codon preference of genes for highly expressed proteins, such as ribosomal proteins and elongation factors. In this study, the values of CAI were calculated using a reference set of ribosomal proteins. Based on the calculated CAI value, 5% of the total genes with extremely high CAI values were regarded as the highly expressed datasets.

Effective number of codons (ENc) was often used to quantify the codon usage bias of a gene. The ENc value of a gene could range from 20 (extreme bias where one codon for each codon family was used) to 61 (all synonymous codons were used randomly). As in the previous report, P_1_, P_2_, and P_3_ were calculated after excluding ATG, TGG, ATA, and the stop codons (TAA, TAG, or TGA) [52]. The value of GC3s was the frequency of G + C at the synonymous third position of sense codons and it was employed to better understand the codon usage variation and compositional constraints (i.e., excluding Met, Trp, and termination codons). The ENc value against GC3s was computed, which was assumed equal to the use of G and C (A and T) in degenerate codon groups. The expected ENc value under random codon usage was calculated for any value of GC3s as below:

(1) ENc = 2 + *s* + 29[*s*^2^ + (1 − *s*)^2^]^−1^

where *s* represents the given GC3s value. If the G + C content at the third position is the only determinant factor that shapes the codon usage, the point of ENc should fall on the standard curve described by Formula (1).

### 4.3. Correspondence Analysis and Cluster Analysis

The correspondence analysis (COA) was used to investigate the major trend in codon usage variation among genes of 12 RA strains. The CDS of each gene was represented as a 59 dimensional vector (excluding ATG, TGG, and the stop codons), and each dimension corresponds to the RSCU value of one sense codon. Since the first two axes, compared to the other axes, would be enough to explain the higher fraction of the variance of the data, genes and codons were plotted on these two axes only [53,54]. In the cluster analysis, RA species were clustered according to their RSCU values by hierarchical methods through measurement of the Squared Euclidean distance.

### 4.4. Software and Statistical Analysis

RSCU, ENc, total G + C genomic content, as well as COA, were calculated by CodonW 1.4 version [55]. The heat map was drawn with HemI (Huazhong University of Science and Technology, Wuhan, Hubei, China) [56] and clustered the RSCU values using an average linkage cluster algorithm. Values of CAI, P1, P2 and P3 were calculated by CAIcal Server [57]. The highly-expressed-gene datasets were interpretation of high-level functions by BlastKOALA [58]. Correlation analysis was performed using the statistical software SPSS 19.0 (IBM, Chicago, IL, USA). Graphs were generated with GraphPad Prism 6.0 (GraphPad Software Inc., La Jolla, CA, USA).

## 5. Conclusions

To summarize, our study reveals that codon usage bias in RA is slightly biased, and there is no significant difference between the strains in codon usage. Natural selection is the main factor that affects codon usage variation in RA. Other factors, such as GC content and gene expression also have an influence on codon usage pattern. In addition, all RA strains have the common highly abundant proteins. To our knowledge, this research is the first work of its kind to report of codon usage analysis in RA, and it gives us a basic understanding of the mechanisms for codon usage bias and gene expression during the evolution of RA. Moreover, this study has provided a basis for further studies on the mechanisms of codon usage that affects the RA strains through evolution.

## Figures and Tables

**Table 1:** The RSCU analysis of the preferred codons, the optimal codons and the rare codons for RA.

Amino Acids	Codon	RSCU ^1^	Amino Acids	Codon	RSCU ^1^
Ala	GCG	0.49	Pro	CCC	0.31
	GCC	0.61		CCG	0.33
	GCU *	**1.66**		CCA *	**1.27**
	GCA *	**1.24**		CCU *	**2.10**
Cys	UGC	0.57	Arg	AGG	0.37
	UGU *	**1.43**		CGC	0.71
Asp	GAC	0.55		CGG	0.19
	GAU *	**1.45**		AGA *	**1.63**
Glu	GAG	0.60		CGA	**1.14**
	GAA	**1.40**		CGU *	**1.96**
Phe	UUC	0.47	Ser	AGC	0.68
	UUU *	**1.53**		UCC	0.46
Gly	GGG	0.66		UCG	0.49
	GGC	0.56		AGU *	**1.32**
	GGU *	**1.36**		UCA	0.80
	GGA *	**1.42**		UCU *	**2.24**
His	CAC	0.80	Thr	ACC	0.89
	CAU *	**1.20**		ACG	0.50
Ile	AUC	0.55		ACA *	**1.20**
	AUU *	**1.41**		ACU *	**1.41**
	AUA	**1.04**	Val	GUG	0.82
Lys	AAG	0.47		GUC	0.18
	AAA *	**1.53**		GUU *	**1.26**
Leu	CUC	0.51		GUA *	**1.74**
	CUG	0.39	Try	UAC	0.62
	UUG	0.68		UAU *	**1.38**
	CUA *	**1.30**	Gln	CAG	0.57
	CUU *	**1.81**		CAA *	**1.43**
	UUA *	**1.32**	Stop	UGA	0.20
Asn	AAC	0.66		UAG	0.61
	AAU *	**1.34**		UAA *	**2.19**
Met	AUG	1.00			

^1^ Average value of RSCU in 12 RA genomes; * represents the optimal codons (*p*-value < 0.01). The preferred codons (RSCU > 1) are in bold.

**Table 2:** Characteristic in the indices of codon bias of RA genes.

RA Strain	GC%	GC3s%	ENc	CAI
ATCC11845	35.42 ± 3.63	26.50 ± 5.75	45.04 ± 5.13	0.616 ± 0.062
RA-CH-1	35.51 ± 3.85	27.05 ± 6.28	45.47 ± 5.15	0.604 ± 0.064
CH3	35.59 ± 3.89	27.07 ± 6.30	45.46 ± 5.17	0.582 ± 0.070
RA-CH-2	35.39 ± 3.62	26.64 ± 5.98	45.19 ± 5.19	0.616 ± 0.064
RA-GD	35.33 ± 3.64	26.67 ± 5.95	45.16 ± 5.20	0.602 ± 0.069
RA-SG	35.40 ± 3.60	26.52 ± 5.17	45.10 ± 5.07	0.609 ± 0.063
RA-YM	35.50 ± 3.58	26.69 ± 5.80	45.15 ± 5.09	0.610 ± 0.063
Yb2	35.34 ± 3.64	26.50 ± 5.74	45.12 ± 5.14	0.613 ± 0.062
RA-JLLY	35.45 ± 3.89	26.91 ± 6.25	45.34 ± 5.21	0.605 ± 0.067
RA153	35.44 ± 3.61	26.57 ± 5.86	45.18 ± 5.18	0.604 ± 0.064
RA17	35.52 ± 3.59	26.55 ± 5.77	45.14 ± 5.22	0.690 ± 0.064
RCAD0122	35.40 ± 3.65	26.69 ± 5.86	45.21 ± 5.14	0.607 ± 0.064

**Table 3:** Orthologous high-level expression genes found in 12 RA strains.

Category	Gene	Proteins	Strains
Ribosome	*rplA*	Large subunit ribosomal protein L1	RA-CH-1, RA-GD, RA17
	*rplB*	Large subunit ribosomal protein L2	+
	*rplD*	Large subunit ribosomal protein L4	+
	*rplE*	Large subunit ribosomal protein L5	+
	*rplF*	Large subunit ribosomal protein L6	+
	*rplL*	Large subunit ribosomal protein L7/L12	+
	*rplI*	Large subunit ribosomal protein L9	+
	*rplJ*	Large subunit ribosomal protein L10	+
	*rplK*	Large subunit ribosomal protein L11	RA-CH-1
	*rplN*	Large subunit ribosomal protein L14	+
	*rplO*	Large subunit ribosomal protein L15	+
	*rplP*	Large subunit ribosomal protein L16	RA-CH-2, CH3, ATCC11845
	*rplQ*	Large subunit ribosomal protein L17	+
	*rplR*	Large subunit ribosomal protein L18	+
	*rplS*	Large subunit ribosomal protein L19	+
	*rplU*	Large subunit ribosomal protein L21	+
	*rplV*	Large subunit ribosomal protein L22	+
	*rplX*	Large subunit ribosomal protein L24	+
	*rpsA*	Small subunit ribosomal protein S1	+
	*rpsB*	Small subunit ribosomal protein S2	+
Ribosome	*rpsC*	Small subunit ribosomal protein S3	+
	*rpsD*	Small subunit ribosomal protein S4	+
	*rpsE*	Small subunit ribosomal protein S5	RA-CH-1, CH3
	*rpsG*	Small subunit ribosomal protein S7	+
	*rpsH*	Small subunit ribosomal protein S8	Except RA17, RA-GD
	*rpsI*	Small subunit ribosomal protein S9	+
	*rpsK*	Small subunit ribosomal protein S11	+
	*rpsO*	Small subunit ribosomal protein S15	+
	*rpsP*	Small subunit ribosomal protein S16	CH3
	*rpsR*	Small subunit ribosomal protein S18	+
Elongation factor	*tuf*	Elongation factor Tu	+
	*fusA*	Elongation factor G	+
	*tsf*	Elongation factor Ts	+
Chaperone	*dnaK*	Molecular chaperone DnaK	Except CH3
	*groEL*	Chaperonin GroEL	+
	*tig*	Trigger factor	+
Enzymes	*acnA*	Aconitate hydratase	+
	*sucC*	Succinyl-CoA synthetase β subunit	+
	*sucD*	Succinyl-CoA synthetase α subunit	+
	*mdh*	Malate dehydrogenase	+
	*gapA*	Glyceraldehyde 3-phosphate dehydrogenase	+
	*ccoP*	Cytochrome c oxidase cbb3-type subunit III	+
	*ccp*	Cytochrome c peroxidase	+
	*atpA*	F-type H^+^-transporting ATPase subunit α	RA-CH-1, RA-CH-2, RA17, ATCC11845
	*atpF*	F-type H^+^-transporting ATPase subunit b	Except CH3
	*pncA*	Nicotinamidase/pyrazinamidase	+
	*ndk*	Nucleoside-diphosphate kinase	+
	*tlpA*	Alkyl hydroperoxide reductase/thiol specific antioxidant/mal allergen	+
	*ppiA*	Peptidyl-prolyl isomerase	+
	*dsrO*	Molybdopterin-containing oxidoreductase	RA-CH-1
	*katE*	Catalase	RA153, ATCC11845, Yb2, RA-SG
	*ahpC*	Peroxiredoxin	Except RA153, RA17
	*sdhB*	Succinate dehydrogenase/fumarate reductase	RA153, RA17, RA-SG, RA-YM
	*eno*	Enolase	+
Enzymes	*nrfA*	Nitrite reductase	RA-CH-2, RA153, RA17, RA-GD
	*dam*	DNA adenine methylase	RA17, ATCC11845, RA-GD
	*tatD*	TatD DNase family protein	RA-CH-1
	*pabC*	4-Amino-4-deoxychorismate lyase	RA-JLLY
	*ald*	Alanine dehydrogenase	RA-JLLY
	*ribBA*	3,4-Dihydroxy 2-butanone 4-phosphate synthase	RA-JLLY
	-	Peptidase s8 and s53 subtilisin kexin sedolisin	+
	-	Peptidase s46	RA17
	-	Putative FAD dependent oxidoreductase	RA17
	-	Septum formation initiator	RA17
	-	Serine protease	ATCC11845
	-	Nodulation protein X acyltransferase 3	ATCC11845
Binding protein	-	Cyclic nucleotide-binding protein	Except RA17
Transport protein	*arac*	Transcriptional regulator	RA17
Apoptosis protein	*cys*	Cytochrome c	Except RA17, RA-JLLY
Structure protein	*gldl*	Gliding motility protein gldl	RA17, ATCC11845
	*ompH*	Outer membrane protein	+
	*ompa/motb*	ompa/motb domain-containing protein	+
	*ftnA*	Ferritin	RA153, ATCC11845, Yb2, RCAD0122
	*hinT*	Histidine triad (HIT) family protein	CH3
	-	Phosphate-selective porin o and p protein	RA-CH-1

+ represents the gene found in all RA strains.

**Table 4:** RA strains used in this study.

Strain	Serotype	Geographic Location	Accession No.	CDS	CDS (>300 bp)	Reference
ATCC11845	6	USA	CP003388.	1941	1764	[42,43]
RA-CH-1	1	Sichuan	CP003787	2187	1953	
CH3	1	Jiangsu	CP006649	2181	1916	[44]
RA-YM	1	Hubei	AENH00000000	2010	1796	[45]
RA-CH-2	2	Sichuan	CP004020	2044	1844	[46]
RA-GD	2	Guangdong	CP002562	1985	1815	[47]
Yb2	2	Jiangsu	CP007204	2021	1877	[48]
RA153	2	Fujian	CP007504	1919	1730	
RA17	ND ^1^	Fujian	CP007503	1656	1613	
RA-SG	ND ^1^	Guangdong	ANGF00000000	2066	1838	[49]
RA-JLLY	ND ^1^	Hubei	LAVB01000000	2089	1858	[50]
RCAD0122	ND ^1^	Guangdong	LUDU00000000	2149	1892	[51]

^1^ ND: Not determined.
